# A conserved graft formation process in Norway spruce and *Arabidopsis* identifies the PAT gene family as central regulators of wound healing

**DOI:** 10.1038/s41477-023-01568-w

**Published:** 2024-01-02

**Authors:** Ming Feng, Ai Zhang, Van Nguyen, Anchal Bisht, Curt Almqvist, Lieven De Veylder, Annelie Carlsbecker, Charles W. Melnyk

**Affiliations:** 1 Department of Plant Biology, Linnean Center for Plant Biology, Swedish University of Agricultural Sciences, Uppsala, Sweden; 2 Department of Organismal Biology, Physiological Botany, Evolutionary Biology Centre and Linnean Centre for Plant Biology, Uppsala University, Uppsala, Sweden; 3 Department of Plant Biotechnology and Bioinformatics, Ghent University, Ghent, Belgium; 4 Center for Plant Systems Biology, VIB, Ghent, Belgium; 5 Skogforsk (The Forestry Research Institute of Sweden), Uppsala Science Park, Uppsala, Sweden; 6 Present Address: College of Life Sciences, Northwest A&F University, Yangling, China

**Keywords:** Plant regeneration, Plant physiology

## Abstract

The widespread use of plant grafting enables eudicots and gymnosperms to join with closely related species and grow as one. Gymnosperms have dominated forests for over 200 million years, and despite their economic and ecological relevance, we know little about how they graft. Here we developed a micrografting method in conifers using young tissues that allowed efficient grafting with closely related species and between distantly related genera. Conifer graft junctions rapidly connected vasculature and differentially expressed thousands of genes including auxin and cell-wall-related genes. By comparing these genes to those induced during *Arabidopsis thaliana* graft formation, we found a common activation of cambium, cell division, phloem and xylem-related genes. A gene regulatory network analysis in Norway spruce (*Picea abies*) predicted that *PHYTOCHROME A SIGNAL TRANSDUCTION 1* (*PAT1*) acted as a core regulator of graft healing. This gene was strongly up-regulated during both spruce and *Arabidopsis* grafting, and *Arabidopsis* mutants lacking *PAT* genes failed to attach tissues or successfully graft. Complementing *Arabidopsis* PAT mutants with the spruce *PAT1* homolog rescued tissue attachment and enhanced callus formation. Together, our data show an ability for young tissues to graft with distantly related species and identifies the PAT gene family as conserved regulators of graft healing and tissue regeneration.

## Abstract (web summary)

Feng and Zhang et al. develop a micrografting technique for conifer trees allowing distant relatives to join. Grafting in conifers uses a similar process to that in eudicots, and by introducing a conifer graft regulator to *Arabidopsis*, grafting and regeneration are enhanced.

## Main

The cutting and joining of different plants during the process of grafting has been practiced for millennia to combine the best properties of two plants. Grafting likely originated as a means for vegetative propagation but now is commonly used to improve stress tolerance, to retain varietal characteristics and to enhance yields. The majority of commercially grafted plants are eudicots, but gymnosperms too are grafted to propagate desirable varieties for forestry breeding programs and for horticulture^1–4^. Gymnosperms evolved approximately 200 million years before angiosperms and, today, dominate many forest environments and have important economic and ecological consequences^4,5^. Despite their widespread prevalence and their ability to be grafted, we have little understanding of how such a process might function in gymnosperms and its relationship to grafting in angiosperms. In conifers, our ability to successfully graft is limited by various factors including grafting techniques, grafting season, pathogen contamination and the relatedness of species^6–8^. Closely related conifer or eudicot species from the same genus normally successfully graft, whereas combinations from different genera often fail, a phenomenon known as graft incompatibility that limits grafting success^9,10^. The mechanistic basis for graft incompatibility remains unclear but might be due to structural weakness, metabolic imbalances or the activation of defence responses^11–13^. However, not all distantly related grafts fail, and inter-genus grafts within the cactus and Solanaceae families are possible^12^. Recently, inter-family grafts were made using *Petunia hybrida* or *Nicotiana benthamiana* where a cell-wall-related *β-1,4-glucanase* gene is important to promote graft attachment^14,15^. Protocols to successfully graft monocots have recently been established using embryonic tissues which allow both inter- and intra-species grafts^16^. Grafting with such small tissues, a process known as micrografting, is increasingly being used as a tool to improve grafting efficiency and also to characterize the process of grafting itself^16–19^.

Work in tomato, *Sedum* and *Arabidopsis* has revealed a dynamic healing process at the eudicot graft junction^17,20,21^. After cutting, cells expand and divide to adhere tissues and fill the wound. Cell-wall components, including pectins, are secreted, and the expression of cell-wall-related genes such as *β-1,4-glucanase* plays an important role in the early stages of graft attachment^14,22^. Cell divisions lead to the formation of callus, a stem-cell-like tissue, at the cut ends that helps seal the wound. In the final stages of graft formation, the callus and surrounding tissues are differentiated to functional phloem tissues, xylem tissues and outer cell layers to resume vascular transport and reform protective barriers. During grafting, thousands of genes are differentially expressed including early activating transcription factors such as *ETHYLENE RESPONSE FACTORs* (*ERFs*), *DNA BINDING WITH ONE FINGER* (*DOF*) and *NAC DOMAIN-CONTAINING PROTEINs* (*ANACs*)^23,24^. These factors play important roles during grafting to promote tissue adhesion, callus formation and vascular differentiation^23–25^. For instance, a gene relevant for grafting, *ERF115*, also controls the replenishment of root stem cells after wounding and is important for successful root tip regeneration and callus formation^26,27^. The regenerative ability of *ERF115* is enhanced by its interacting partner *PHYTOCHROME A SIGNAL TRANSDUCTION1 (PAT1). PAT1*, along with two other *GIBBERELLIC-ACID INSENSITIVE, REPRESSOR OF GA1, and SCARECROW* (*GRAS*) transcription factors, *SCARECROW-LIKE5* (*SCL5*) and *SCL21*, are important for root tip regeneration and cell death recovery^26,28^. *ERF115* also plays an important role during wounding to enhance auxin sensitivity by activating *AUXIN RESPONSE FACTOR5* (*ARF5)*^29^. Auxin is important for graft formation as blocking auxin transport inhibits the ability of *Arabidopsis* and rice grafts to heal, whereas reducing auxin response below the graft junction inhibits *Arabidopsis* graft healing^16,17,30^. Other early activators during grafting include *WUSCHEL-RELATED HOMEOBOX13* (*WOX13*) and *WOUND INDUCED DEDIFFERENTIATION1 (WIND1)* that are important for callus formation at the site of cutting^31,32^. Thus, work in *Arabidopsis*, rice and *Nicotiana* has identified numerous factors that are activated early and contribute to attachment, callus formation and vascular differentiation during grafting.

In this Article, we investigated the process of graft formation in several widespread and commercially relevant conifer species. We developed an efficient and practical grafting method using young conifer plants that allowed graft junctions to rapidly heal and permitted several inter-species and inter-genus graft combinations to successfully form. We used this method to characterize graft healing and discovered a common graft formation pathway in Norway spruce (*Picea abies*) and *Arabidopsis* that involved cell division, vascular differentiation and the up-regulation of cell-wall- and auxin-related genes. We additionally identified that *PAT1* up-regulation is common in *Arabidopsis* and *Picea* *abies* grafting and that this gene appears to have a conserved role in wound healing between gymnosperms and eudicots.

## Results

### A new method for conifer grafting

**Fig. 1:**
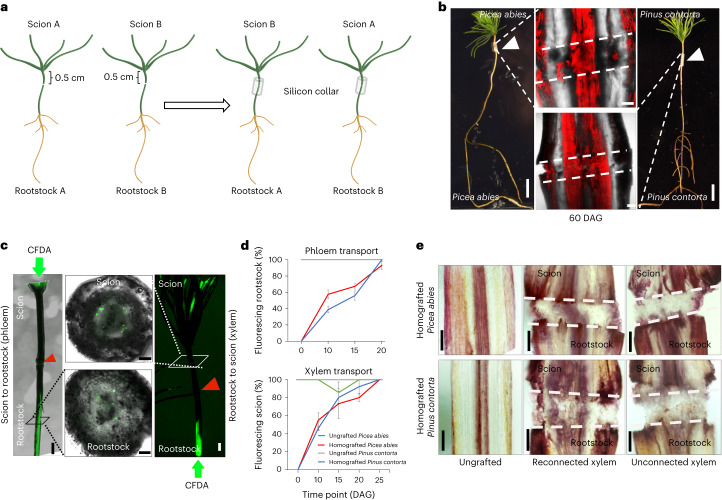
Micrografting dynamics in conifers. **a**, Cartoons showing the micrografting method used in conifers. Ten- to 12-day-old scions and rootstocks from different plants are grafted with silicon collars. **b**, Homografted *Picea* *abies* (left, *n* = 48) and *Pinus contorta* (right, *n* = 40) at 60 DAG. Scale bars, 1 cm. White triangles indicate the graft junction. Middle panels: confocal images of the vascular anatomy at the graft junction. Three to four plants per combination per replicate, two biological replicates. Scale bars, 100 µm. **c**, Phloem and xylem transport assays in conifers involving CFDA application to the scion (phloem) and rootstock (xylem) monitored the appearance of fluorescence in rootstock or scion, respectively, consistent with phloem or xylem transport. Scale bars, 1 mm. Hand-section stems above or below the graft junction confirmed vascular transport 15 DAG. Scale bars, 100 µm. *n* = 11 plants per replicate, 3 biological replicates. **d**, Phloem and xylem reconnection rates in *Picea* *abies* and *Pinus contorta* grafts as measured by CFDA transport (mean ± s.d. of 3 biological replicates, *n* = 8–21 plants per time point per replicate). **e**, Xylem staining with basic fuchsin in grafted *Picea* *abies* and *Pinus contorta*. Plants were from **d**, and grafting success was measured by the presence or absence of CFDA in scions at 15 DAG. *n* = 3–4 plants per combination per replicate, 3 biological replicates. Scale bars, 20 µm. Source data

**Extended Data Fig. 1:**
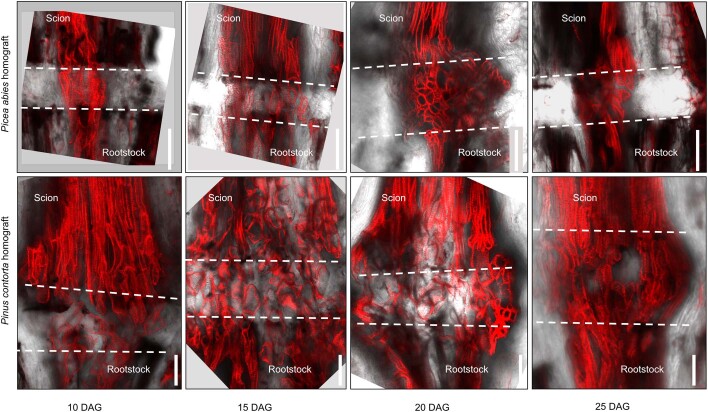
Graft junction healing dynamics in homografted *Picea abies* and homografted *Pinus contorta*. Confocal images of xylem anatomy taken 10 days after grafting (DAG), 15 DAG, 20 DAG or 25 DAG. Plants were from (Fig. 1d) and grafting success was measured by the presence or absence of CFDA in scions after grafting. Xylem was stained with basic fuchsin. n = 4–6 plants per combination per replicate, 2 biological replicates. Scale bars, 100 µm.

Previous conifer grafting methods were limited by various factors including techniques, grafting season, temperature and contamination^3,6,7^. To improve graft formation rates and the ease of grafting, we developed a micrografting method using 10- to 12-day-old spruce (*Picea*) and pine (*Pinus*) seedlings. Plants were excised in the hypocotyl region, and scions and rootstocks from different plants were attached tightly together using a silicon collar (Fig. 1a,b). With practice, one person could perform 50 grafts per hour with >90% success rates (Table 1), a substantial improvement over traditional conifer grafting methods in which upwards of 120 grafts per day are done^7^. To monitor the dynamics of graft healing, we grafted *Picea* *abies* to *Picea* *abies*, and *Pinus contorta* (Lodgepole pine) to *Pinus contorta*, and treated the scion and rootstock with carboxyfluorescein diacetate (CFDA), a dye used for testing vascular connectivity in grafted *Arabidopsis* and rice^16,17^. Ten days after grafting (DAG), we observed that nearly half of plants transported CFDA from the scion to the rootstock, consistent with resumption of shoot-to-root transport through the phloem (Fig. 1c,d). Similarly, nearly half of plants at 10 DAG showed movement of CFDA from rootstock to the scion consistent with resumption of root-to-shoot transport through the xylem (Fig. 1c,d). By 20–25 DAG, nearly all individuals showed transport dynamics consistent with phloem and xylem connectivity. As a second test of vascular reconnection, we stained hand sections from the graft junction with basic fuchsin to assess the presence of xylem-associated lignin. Successful grafts showed xylem connections across the junction, whereas unconnected plants showed little xylem staining and only callus formation at the junction (Fig. 1e and Extended Data Fig. 1). Two months after grafting, the grafted spruce and pine showed normal growth, well-healed junctions and survival rates of 90–100% (Fig. 1b and Supplementary Table 1). Thus, our technique was an efficient and practical method for grafting young conifers that allowed the graft junction to rapidly form xylem and phloem connections after grafting.

**Table 1:** Graft success rate in different conifer combinations after 2.5 years of growth

Scions	*Picea abies*		*Pinus sylvestris*		*Pinus contorta*		*Larix* hybrid		*Larix sibirica*	
Rootstocks	Ratio	%	Ratio	%	Ratio	%	Ratio	%	Ratio	%
*Picea abies*	45/48	93.8	11/56	19.6	17/55	30.9	NP		3/9	33.3
*Pinus sylvestris*	2/56	3.6	73/73	100	37/38	97.4	0/10	0	0/29	0
*Pinus contorta*	1/55	1.8	36/40	90	40/40	100	NP		NP	
*Larix* hybrid	NP		0/8	0	NP		7/8	87.5	3/6	50
*Larix sibirica*	1/6	16.7	0/15	0	NP		5/8	62.5	7/9	77.8

Note: NP, not performed.

Source data

### Micrografting allows heterograft success

**Fig. 2:**
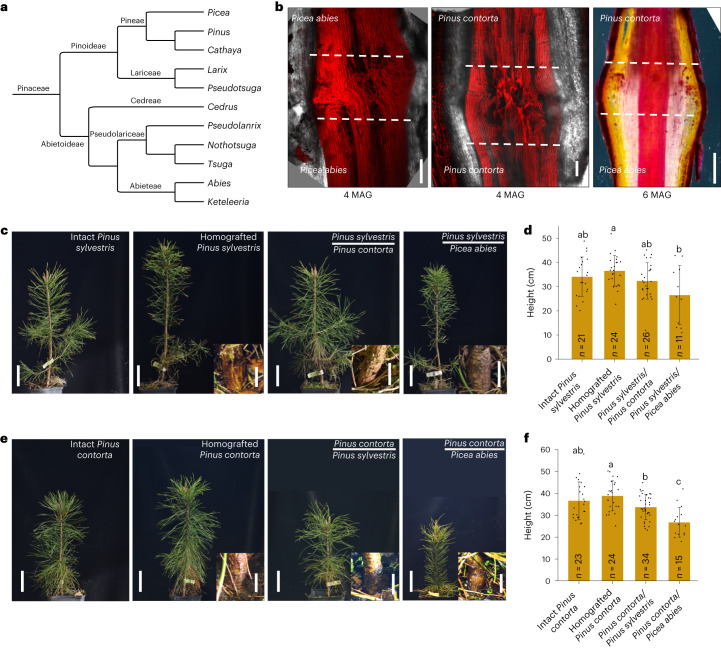
Micrografting enables inter-genus grafting. **a**, Phylogenetic tree showing the intergeneric relationships of Pinaceae based on published data^34^. **b**, Junction anatomy of homografted or heterografted *Pinus contorta* and *Picea abies* 4 or 6 months after grafting (MAG). Scale bars, 100 µm for images at 4 MAG, 1 mm for image at 6 MAG. *n* = 3 plants per combination per replicate, 2 biological replicates. **c**,**e**, Representative images of homografted or heterografted *Pinus sylvestris*, *Pinus contorta* and *Picea abies* 2.5 years after grafting. Intact (non-grafted) *Pinus sylvestris* and *Pinus contorta* were used as controls. *Pinus sylvestris* was used as scions in (**c**), and *Pinus contorta* was used as scions in (**e**). Scale bars, 10 cm. Inserts show the graft junctions. Scale bars, 1 cm. **d**,**f**, Height of 2.5-year-old intact and grafted *Pinus sylvestris*, *Pinus contorta* and *Picea abies* (mean ± s.d., *n* = 11–34 plants per combination). *Pinus* *sylvestris* was used as scions in (**d**), and *Pinus contorta* was used as scions in (**f**). Different letters above the bars represent *P* < 0.05. One-way ANOVA, with Tukey’s post hoc test. *P* values are shown in source data. Source data

**Extended Data Fig. 2:**
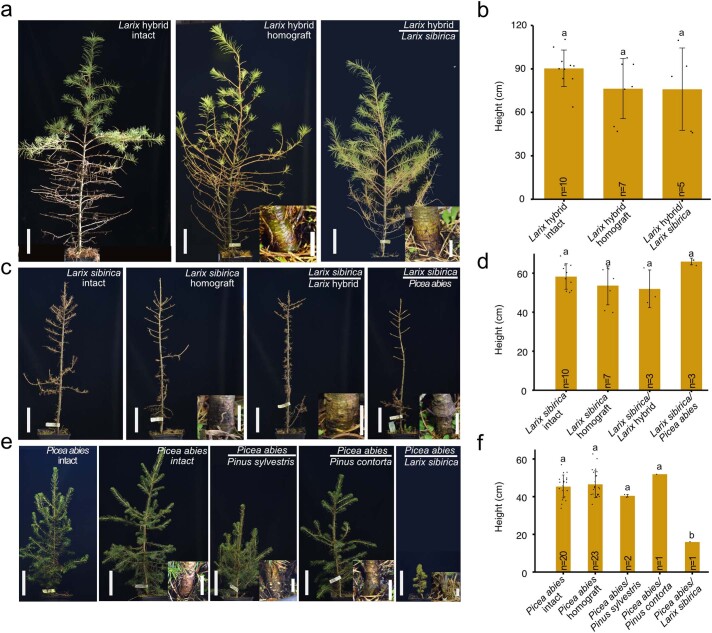
Inter-genus micrografting. **a**, **c** and **e**, Representative images of homografted or heterografted *Larix* hybrid, *Larix sibirica, Picea abies*, *Pinus sylvestris* and *Pinus contorta*. Images were taken in November when *Larix* were entering dormancy and loosing their needles. Intact (non-grafted) *Larix* hybrid, *Larix sibirica* and *Picea abies* were used as controls. Scale bars, 10 cm. Inserts show the graft junctions. Scale bars, 1 cm. **b**, **d** and **f**, Height of 2.5 years old intact and grafted *Larix* hybrid, *Larix sibirica, Picea abies*, *Pinus sylvestris* and *Pinus contorta* (mean ± SD from 1–23 plants per combination). Different letters represent *p* < 0.05. One-way ANOVA, with Tukey’s post-hoc test. *p* values are shown in source data. Source data

Previous studies found that grafting success decreases and incompatibility increases as conifer species become more distantly related, and inter-genus grafts are generally not possible^3,9,33^. We therefore tested whether our micrografting method overcame this limitation. As the Pinaceae family shows the closest relatedness between *Picea* and *Pinus* (Fig. 2a)^34^, we first tested inter-genus grafting between *Picea* and *Pinus* species. Two months after grafting, species grafted to themselves (homografts) or different species (heterografts) had high survival rates varying between 70% and 100% depending on the genotype (Supplementary Table 1). We assessed xylem connectivity in *Pinus contorta*/*Picea abies* heterografts (scion/rootstock notation) and found xylem connectivity was similar to homografted plants (Fig. 2b). Incompatibility in woody species can develop several years after grafting^33^, so we moved plants to soil for long-term observation. Survival rates of homografted plants remained high 2.5 years after grafting (90% to 100%), but heterografted *Picea*–*Pinus* combinations showed lower survival rates (Table 1). *Picea abies* scions grafted to *Pinus sylvestris* (Scots pine) and *Picea* *abies* scions grafted to *Pinus contorta* rootstocks had very low survival rates (3.6% and 1.8% viable, respectively). However, survival rates were substantially higher when *Picea* *abies* rootstocks were grafted to *Pinus sylvestris* or *Pinus contorta* scions (19.6% and 30.1% viable, respectively) (Table 1). Successful grafts showed good growth, although some heterografted combinations were shorter and showed swelling at the graft junction. Heterografted plants without swelling had similar heights to intact plants (Fig. 2c–f and Supplementary Table 2). *Picea* *abies* rootstocks also appeared to reduce the needle length of the *Pinus* scions (Fig. 2c,e). Next, we tested two species from the Pinaceae genus *Larix* in heterografts (Extended Data Fig. 2). *Larix sibirica*/*Pinus sylvestris* combinations did not survive, whereas *Larix sibirica*/*Picea* *abies* had three of nine plants grow well and appear to overcome graft incompatibility but with swelling at the graft junction (Table 1 and Extended Data Fig. 2). One of six *Picea* *abies*/*Larix sibirica* survived, but it showed poor growth (Table 1 and Extended Data Fig. 2). Our results suggested that micrografting allowed several inter-species and inter-genus grafts to successfully form and that *Picea* *abies* rootstocks enabled long-term grafting success with divergent scions.

### Grafting activates vascular and cell-division-associated genes

**Fig. 3:**
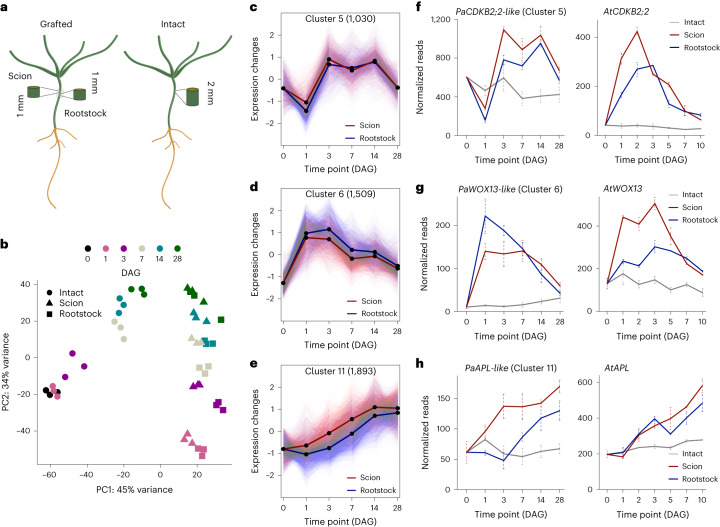
Transcriptome dynamics during *Picea abies* graft healing. **a**, Schematic diagram showing where *Picea abies* tissues 1 mm above or 1 mm below the graft junction were collected as scion and rootstock material, respectively, for transcriptome analysis. TF, transcription factor. **b**, PCA of the gene expression data from *Picea abies* graft healing transcriptomes. The two principal components (PC1 and PC2) explained 79% of the total variation in the grafting transcriptomes and showed correlations with graft healing along PC1 and with time along PC2. Colours indicate the different DAG; shapes indicate different tissues. Shown are data from three biological replicates per tissue per time point. **c**–**e**, Clustering analysis of transcriptional dynamics during graft healing for clusters 5 (**c**), 6 (**d**) and 11 (**e**). Lines indicate the average of DEGs in scion or rootstock. Dots indicate DAG. The number in the brackets represents the number of genes in the cluster. **f**–**h**, Expression profiles for select *Picea abies* genes belonging to clusters 5 (**f**), 6 (**g**) and 11 (**h**). Cluster number is indicated, and the mean (±s.d.) from three biological replicates per time points per tissue is shown. *Arabidopsis* homolog expression data are plotted and taken from previously published transcriptome data^23^ with a mean (±s.e.m.) from 2 biological replicates per tissue per time point. Source data

**Extended Data Fig. 3:**
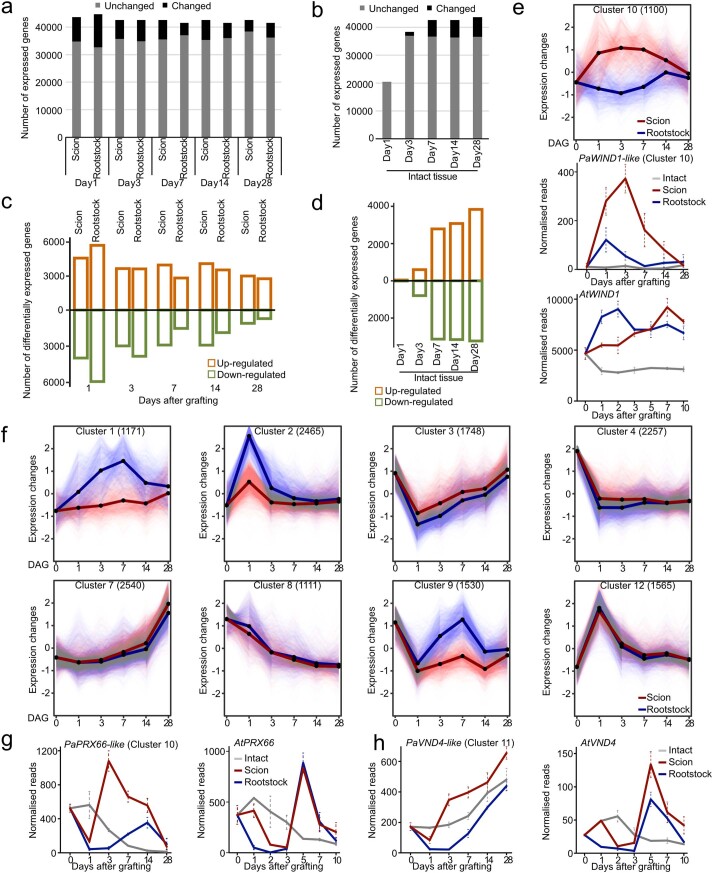
Differentially expressed genes during *Picea abies* grafting. **a**, **b**, Total differentially or non-differentially expressed genes in grafted *Picea abies* scions, rootstocks or intact tissues. Differential expression was determined by comparing to intact samples at the same time point (**a**) or by comparing to the day zero intact sample (**b**). **c**, **d**, Total number of upregulated and downregulated genes in scion, rootstock or intact *Picea abies* tissues. Differential expression was determined by comparing to intact samples at the same time point (**c**) or by comparing to the day zero intact sample (**d**). **a-d**, 3 biological replicates per tissue per treatment. **e-h**, Clustering analysis of transcriptional dynamics during graft healing. Lines indicate the average of differentially expressed genes in scion or rootstock and the expression profiles for selected *Picea abies* genes plotted over grafting. Dots indicate days after grafting (DAG). The number in the brackets represents the number of genes in the cluster. Cluster number is indicated and the mean (± SD) from 3 biological replicates per time points per tissue is shown. *Arabidopsis* homolog expression data are plotted and taken from published transcriptome data^23^ with a mean (± SEM) from 2 biological replicates per tissue per time point is shown. Source data

To understand the process behind conifer grafting and to identify the genes differentially expressed, we generated RNA sequencing (RNA-seq) libraries from both ungrafted (intact) and grafted *Picea abies* above and below the graft junction at 0, 1, 3, 7, 14 and 28 DAG (Fig. 3a). A principal component analysis (PCA) showed samples largely clustered by tissues type and time point with a close correlation between scion and rootstock samples (Fig. 3b). Intact and grafted samples had similar numbers of expressed genes, yet there was an increase in differential expression in grafted samples particularly at 1 DAG and 3 DAG compared to intact controls (Extended Data Fig. 3a–d and Supplementary Table 3). To analyse common patterns of gene expression in grafted tissues, we used Mfuzz (version 3.15) to group differentially expressed genes (DEGs) into 12 clusters (Fig. 3c–e and Extended Data Fig. 3e,f). These included genes showing both scion and rootstock up-regulation (clusters 5, 6, 7, 11, 12), scion-specific up-regulation (cluster 10), rootstock-specific up-regulation (clusters 1, 2, 9) and down-regulation in scion and rootstock (clusters 3, 4, 8). A Gene Ontology (GO) analysis on the clusters revealed enrichment of wounding and defence-related processes in cluster 2, enrichment of cell-cycle-related processes in cluster 5 and enrichment in cell-wall- and xylem-related processes in cluster 10 (Supplementary Table 4). Within these clusters, we searched for homologs of previously described grafting-related genes^23,31,35^ to better understand how conifers graft. Cluster 6 contained early activating genes in the scion and rootstock including a wounding-related *PaWOX13-like* gene^31^(Fig. 3d,g). Cluster 10 contained early activating scion-specific genes including a wounding-related *PaWIND1-like* gene (Extended Data Fig. 3e). Cluster 5 contained genes that slightly later increased in both rootstock and scion including cell-cycle-related genes such as *PaCDKB2;2-like* (Fig. 3c,f) (Supplementary Table 4). A phloem-related *PaAPL-like*, xylem-related *PaPRX66-like* and *PaVND4-like* genes had intermediate and late activation dynamics (Fig. 3e,h and Extended Data Fig. 3g,h). To compare gene expression profiles in grafted conifers and eudicots, we analysed the grafting transcriptomics dataset from *Arabidopsis*^23^ and found the expression of several *Picea abies* gene homologs had similar expression patterns in *Arabidopsis* (Fig. 3f–h, Extended Data Fig. 3e,g,h). Thus, there appeared to be consistent activation of homologous genes during *Arabidopsis* and *Picea abies* grafting suggesting a conserved grafting process involving wound response, followed by cell division, and phloem and xylem differentiation.

### Auxin responses increase and correlate with cell-wall-related gene expression

**Fig. 4:**
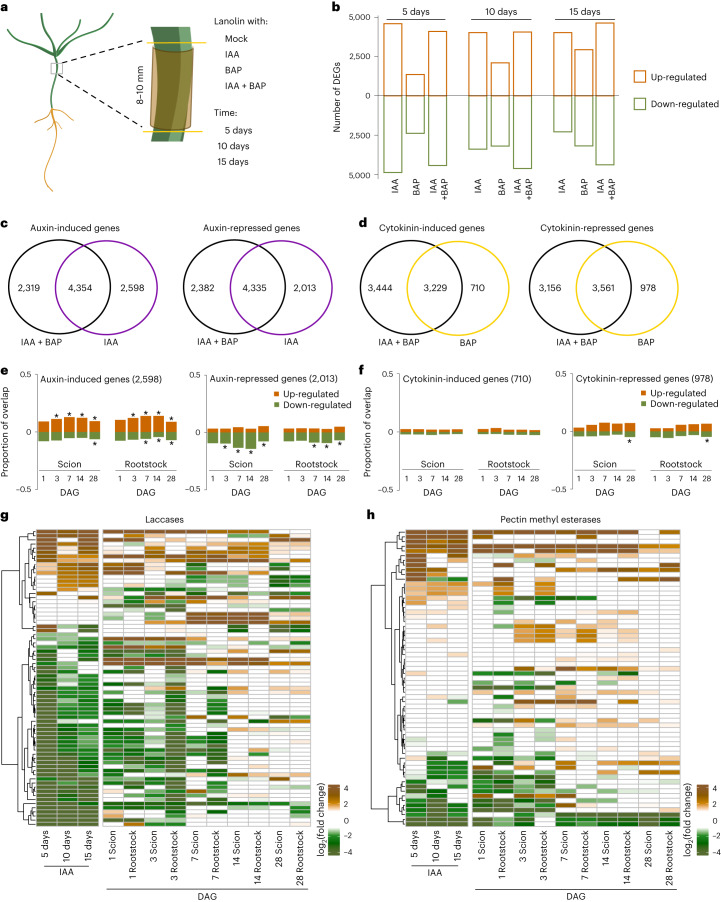
*Picea abies* grafting activates auxin-responsive and cell-wall-related gene expression. **a**, Schematic diagram showing the regions collected for the hormone transcriptomes in *Picea abies*. About 50 mM auxin (IAA), 135 µM cytokinin (BAP) or auxin plus cytokinin (IAA + BAP) was applied, and tissues were collected 0 day, 5 days, 10 days and 15 days after treatment. **b**, The distribution of DEGs responding to hormone treatments. **c**,**d**, Venn diagrams showing the auxin-responsive genes (**c**) and cytokinin-responsive genes (**d**) differentially expressed by all three time points in the various treatments. **e**,**f**, An overlap analysis presented as a ratio of 1.0 for genes differentially expressed in response to hormones (auxin (**e**) and cytokinin (**f**)) and genes differentially expressed during *Picea abies* grafting (Extended Data Fig. 3c). Asterisks indicate statistically significant overlap between hormone response and graft healing. Significance was calculated by a Fisher test (one sided) with FDR adjustment. *P < 0.05. *P* values are shown in source data. **g**,**h**, Heat map showing the fold changes of putative laccase genes (**g**) and putative pectin methyl esterases (PMEs) (**h**) in graft healing and auxin datasets. Source data

**Extended Data Fig. 4:**
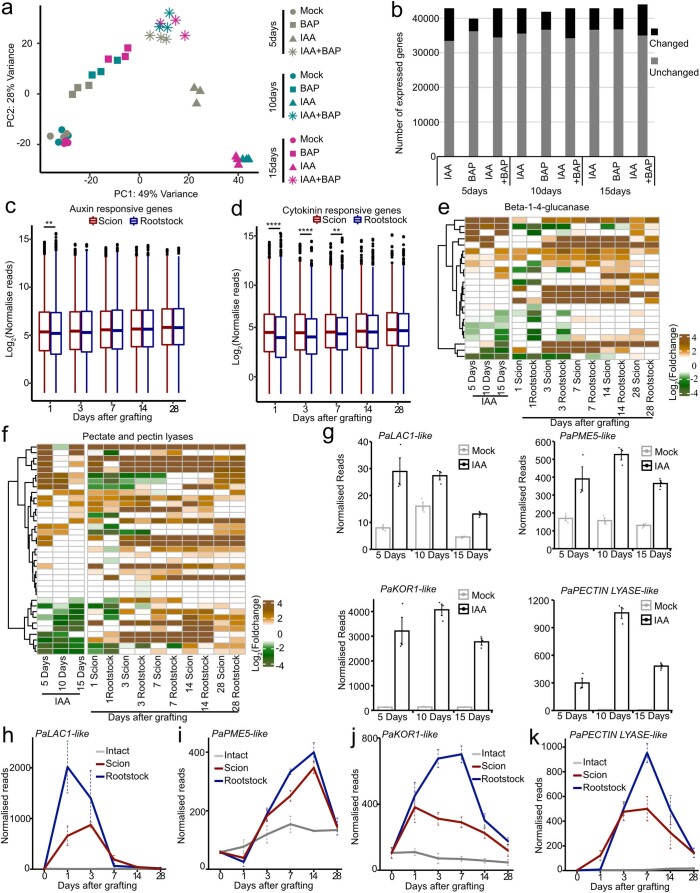
Auxin, cytokinin and cell wall related genes expression. **a**, Principal component analysis of the gene expression data during hormone treatments. Colors indicate different time points and shapes indicate different treatments including auxin (IAA), cytokinin (BAP) and auxin plus cytokinin (IAA+BAP). Shown are data from 3 biological replicates per treatment per time point. **b**, Total differentially or non-differentially expressed genes in hormone treatments at different time points. **c**, Average expression of auxin responsive genes during graft healing (Fig. 4c). **d**, Average expression of cytokinin responsive genes during graft healing (Fig. 4d). Asterisks indicate statistically significant differences between scion and rootstock tissues. **p<0.05 was calculated using Wilcoxon’s test (two-sided) with FDR adjustment. *p* values are shown in source data. Box plots show the 25% quantile, median (line) and 75% quantile of the reported values, respectively. The whiskers correspond to 1.5× the interquartile range. Black points indicate outliers. **e**, **f**, Heatmap showing the fold changes of putative beta-1–4-glucanase genes or putative pectate and pectin lyase genes in graft healing or auxin datasets. **g–k**, Expression profiles of *PaLAC1-like* (MA_75861g0010), *PaPME5-like* (MA_10267810g0010), *PaKOR1-like* (MA_69345g0010) and *PaPECTIN LYASE-like* (MA_6752784g0010) in the auxin treatment datasets (**g**) or grafting datasets (**h-k**). Data are presented as mean (± SD) from 3 biological replicates per tissue per time point. Source data

Auxin and cytokinin play important roles during vascular formation^36,37^. We explored whether they were relevant for *Picea abies* graft formation. Auxin and cytokinin-responsive genes in the hypocotyl are not well described in *Picea*, so we first treated 2-week-old seedlings with a synthetic cytokinin 6-benzylaminopurine (BAP), auxin (indole-3-acetic acid (IAA)) or BAP + IAA for 0 day, 5 days, 10 days and 15 days (Fig. 4a). We collected 8–10 mm of treated *Picea abies* hypocotyl tissues and used these for RNA-seq analyses. A PCA grouped samples together largely based on hormone treatment rather than time point (Extended Data Fig. 4a). Looking at the individual time points, we found several thousand genes were auxin responsive or responded to both hormones, while a slightly lower number responded to cytokinin (Fig. 4b–d, Extended Data Fig. 4b and Supplementary Table 5). To focus on genes specifically induced by auxin or cytokinin, we looked for genes induced at all three time points but induced only by the presence of cytokinin or auxin alone. We found auxin induced 2,598 genes and repressed 2,013 genes, which we defined as auxin-responsive genes. Cytokinin induced 710 genes and repressed 978 genes, which we defined as cytokinin-responsive genes (Fig. 4c,d). In grafted scions and rootstocks, the average expression of these hormone-responsive genes was similar (Extended Data Fig. 4c,d), but when comparing genes differentially expressed by grafting and hormone treatment, we saw an enrichment in auxin-induced genes up-regulated in scions and rootstocks, and an enrichment in auxin-repressed genes down-regulated in scions and rootstocks, from 3 DAG onwards (Fig. 4e). Cytokinin-induced genes showed little enrichment in the scion or rootstock but cytokinin-repressed genes showed enrichment at later time points particularly in the rootstock (Fig. 4f).

Auxin response and cell wall modifications are important for successful graft formation^14,24,38^ so we assessed whether auxin might affect cell-wall-related gene expression. Many putative laccase, pectin methyl esterase (PME), beta-1-4-glucanase and pectate/pectin lyase genes showed differential expression both after exogenous auxin treatment and after grafting including *PaLAC1-like*, *PaPME5-like*, *PaKOR1-like* and *PaPECTIN LYASE-like* (Fig. 4g,h and Extended Data Fig. 4e–k). In particular, there was a substantial overlap between laccases and PMEs affected by both auxin treatment and grafting (Fig. 4g,h). These results showed that grafting induced an auxin response at the junction, and this correlated with the activation of cell-wall-related genes at the graft junction.

### A conserved *PAT1* gene family promotes graft healing

**Fig. 5:**
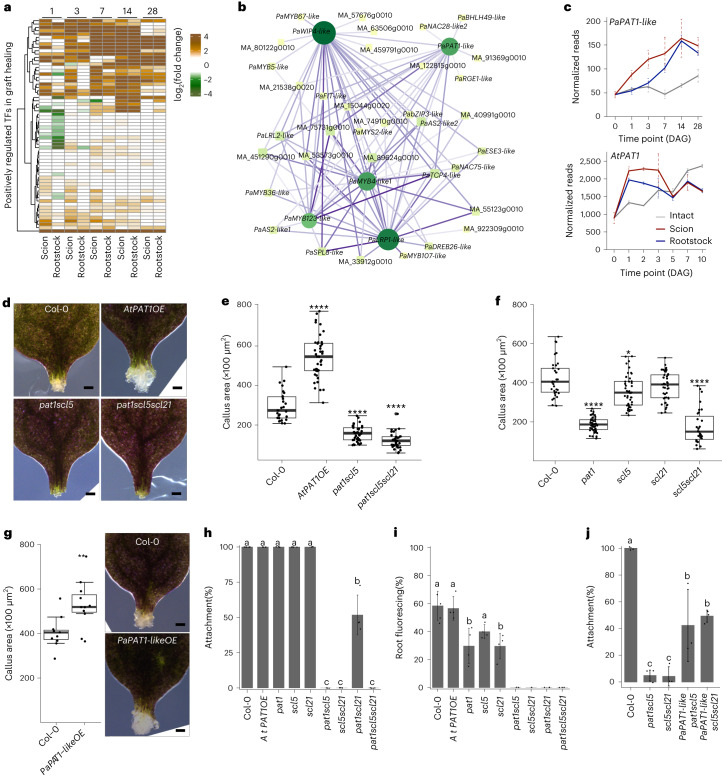
*PAT1*-related genes promote graft healing. **a**, Heat map of positively regulated transcriptional factors during graft healing. **b**, Regulatory connections of the top five core transcriptional factors based on a gene regulation network of transcription factors activated by grafting. The size and colour of nodes indicate the number of edge connections. The edge colour indicates the value of correlation. **c**, *PaPAT1-like* and *AtPAT1* expression during graft formation in *Picea abies* and *Arabidopsis*. The mean (±s.d.) from two to three biological replicates is shown. **d**, Callus formation from cut *Arabidopsis* petioles overexpressing *PAT1* (*AtPAT1OE*) (*n* = 40) or mutants for *pat1scl5* (*n* = 41) or *pat1scl5scl21* (*n* = 35). Scale bars, 100 µm. Two biological replicates. **e**, Callus area in wounded petiole explants in Col-0 (*n* = 28), *AtPAT1OE* (*n* = 40), *pat1scl5* (*n* = 41) or *pat1scl5scl21* (*n* = 35). Two biological replicates. **f**, Callus area in wounded petiole explants in Col-0 (*n* = 33), *pat1* (*n* = 52), *scl5* (*n* = 46), *scl21* (*n* = 46) and *scl5scl21* (*n* = 31). Three biological replicates. In panels **e** and **f**, significance was calculated using Wilcoxon’s test (two-sided) with FDR adjustment, compared with Col-0. Dots indicate individual samples, *P < 0.05, **** *P* < 0.0001; see source data for *P* values. **g**, Images and quantifications of callus formation from cut petioles of Col-0 (*n* = 10) and *PaPAT1-like* overexpression (*n* = 11). Scale bars, 100 µm. Dots indicate individual samples, two-sided Student’s *t*-test; ** *P* < 0.01. Two biological replicates. See source data for *P* values. **h**,**i**, Attachment (**h**) and phloem reconnection (**i**) rates of homografted Col-0, *AtPAT1OE*, *pat1*, *scl5*, *scl21*, *pat1scl5*, *scl5scl21*, *pat1scl21* and *pat1scl5scl21*. **j**, Graft attachment rates of Col-0 and *PaPAT1-like* transformed in *pat1scl5* and *scl5scl21*. In panels **h**–**j**, the mean (±s.d.) from 3 to 6 biological replicates with 10–17 plants per time point per experiment is shown. One-way ANOVA, with Tukey’s post hoc test. Different letters above the bars represent *P* < 0.05. *P* values are shown in source data. Source data

**Extended Data Fig. 5:**
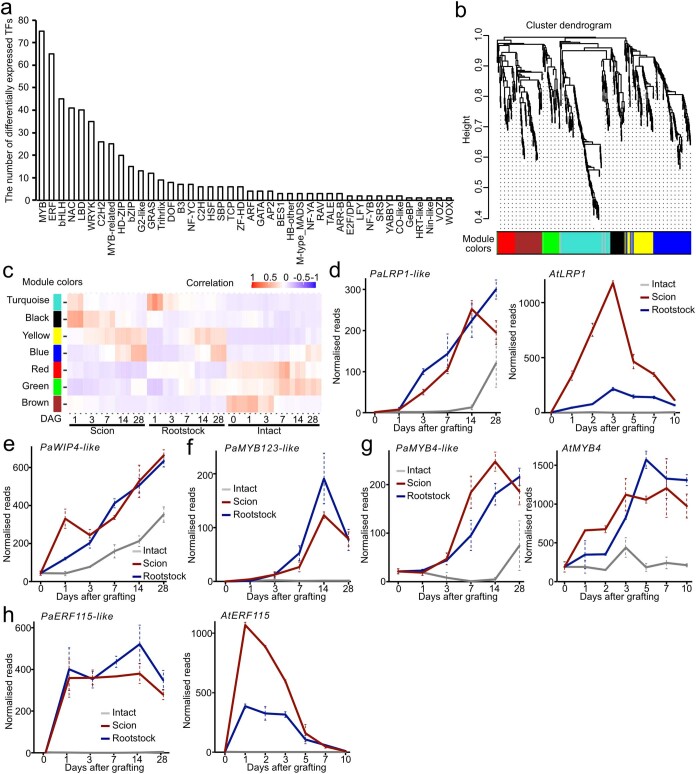
Transcription factor analysis during graft formation. **a**, Bar plot showing the distribution of all differentially expressed transcriptional factors during graft healing. X-axis indicates the different families of transcription factors. Y-axis indicates the number of differentially expressed TFs during spruce graft formation. **b**, Hierarchical cluster tree showing co-expression modules identified using WGCNA. Colors indicate different clusters. **c**, The association between modules and graft healing. Each row corresponds to a module, labeled with a color as in (**b**). Each column corresponds to the grafted samples (scion and rootstock) and intact samples. The color of each cell indicates the correlation coefficient between the module and samples. **d–g**, Expression profiles for the candidate core transcription factor genes during *Picea abies* grafting including *PaLRP1-like* (MA_2299g0010), *PaWIP4-like* (MA_29238g0010), *PaMYB123-like* (MA_10048467g0010) and *PaMYB4-like* (MA_316475g0010). **d** and **g** show the homologs of *PaLPR1-like* and *PaMYB4-like* in the *Arabidopsis* graft formation datasets^23^. **h**, Expression profiles of *PaERF115-like* (MA_10274g0010) and *AtERF115* during graft healing. **d-h**, Data are presented as mean (± SD) from 3 biological replicates per tissue per time points in *Picea abies* and 2 biological replicates per tissue per time point in *Arabidopsis*. Source data

**Extended Data Fig. 6:**
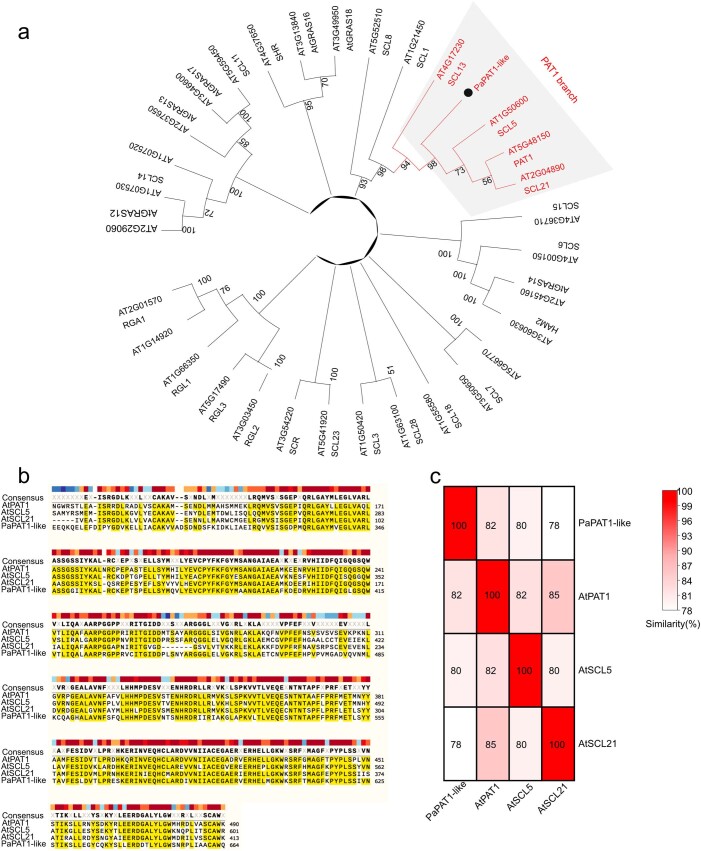
Phylogenetic analysis and amino acid alignment of *Arabidopsis* AtPAT1 family proteins and *Picea abies* PaPAT1-like proteins. **a**, Phylogenetic analysis of *Arabidopsis* GRAS transcription family proteins and *Picea abies* PaPAT1-like proteins. Red text indicates PAT1 family proteins. **b**, Amino acid alignment of the C-terminal of AtPAT1, AtSCL5, AtSCL21 and PaPAT1-like. The consensus sequences are highlighted in yellow. **c**, Heatmap showing the amino acid similarity in percentages for PAT1 family proteins.

**Extended Data Fig. 7:**
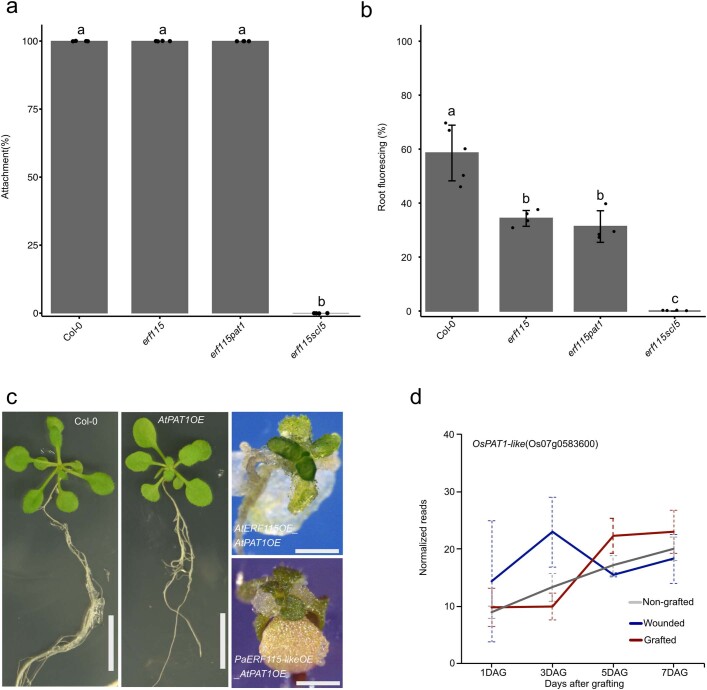
*ERF115* and *PAT1* are important for graft healing and regeneration. **a**, Graft attachment rates in *Arabidopsis* Col-0, *erf115*, *erf115pat1* and *erf115scl5*. **b**, Phloem reconnection rates in *Arabidopsis* Col-0, *erf115*, *erf115pat1* and *erf115scl5*. a and b, Data are presented as mean (± SD) from 4–5 biological replicates with 10–17 plants per time points per treatment. One-way ANOVA, with Tukey’s post-hoc test. *P* values are shown in source data. Different letters represent *p* < 0.05. **c**, Images showing three weeks old Col-0, *AtPAT1OE*, *AtERF115OE AtPAT1OE* and *PaERF115-likeOE AtPAT1OE*. Scale bars for left and middle panels, 1 cm. Scale bars for right panels, 1 mm. **d**, Rice *OsPAT1-like* (Os07g0583600) expression profile in grafted rice adapted from published data^16^ (n = 3 biological replicates). Source data

**Extended Data Fig. 8:**
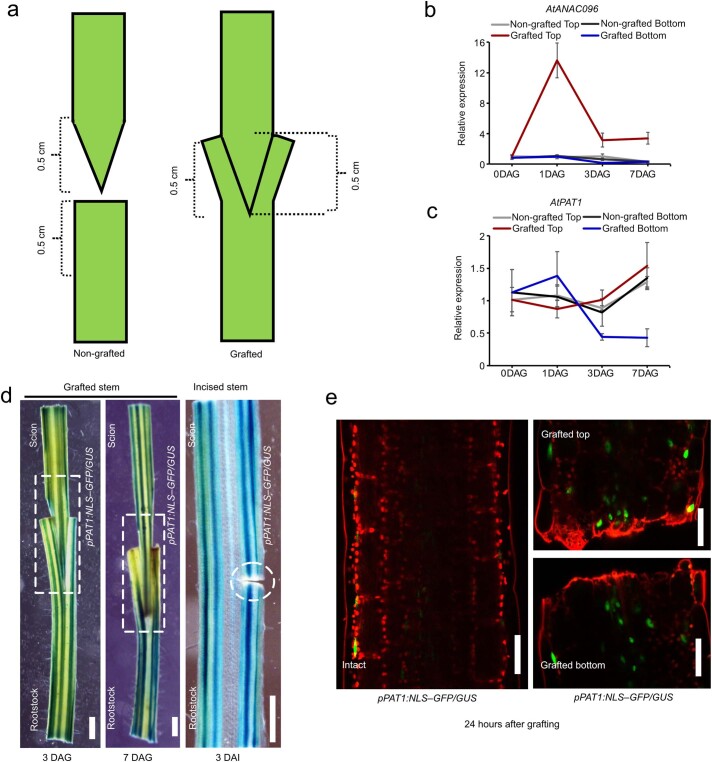
*AtPAT1* expression at inflorescence stem graft junctions and incised stems. **a**, Schematic diagram showing the regions from the inflorescence graft junction for RNA extraction. 0.5 cm stem tissues collected from the scion and rootstock in grafted plants. A similar size and shape tissue was collected from intact plants as controls. **b, c**, Relative gene expression of *AtANAC096* and *AtPAT1* was analyzed by qRT-PCR in grafted plants at 0, 1, 3, 7 days after grafting (DAG). The mean (± SD) is shown from 3–6 biological replicates, 3 plants were pooled together per time point per treatment. **d**, Histochemical analysis of *pPAT1:NLS–GFP/GUS* at 3 or 7 DAG or days after incision (DAI). Left or middle panel is a longitudinal section through the graft junction (junctions indicated by a white rectangle). Right panel is a longitudinal section from an incised stem (wounding site is indicated by a white dashed circle). 2 biological replicates with 4 plants per treatment. Scales bars, 1 mm. **e**, *pPAT1:NLS–GFP/GUS* is activated at the grafted top and bottom 24 hours after grafting (n = 10), intact plant (n = 9) served as a control. Scale bars, 100 µm. 2 biological replicates. Source data

To further explore the transcriptional regulation of *Picea abies* graft formation, we identified differentially expressed transcription factors and mapped their abundance according to transcription factor gene families (Extended Data Fig. 5a). We performed a weighted correlation network analysis (WGCNA), clustered these transcription factors according to their expression patterns in the grafting transcriptomes and defined seven modules (Extended Data Fig. 5b,c). One module, represented in yellow (Extended Data Fig. 5c), showed differential expression specifically in scions and rootstocks but not in intact plants. Using the expression patterns of the genes in the yellow module, we generated a gene regulatory network (Fig. 5a,b). Most transcription factors in this module were up-regulated during grafting, and five in particular, *PaPAT1-like*, *PaWIP4-like*, *PaMYB4-like*, *PaLRP1-like* and *PaMYB123-like*, were highly up-regulated and appeared to act as hubs of the regulatory network (Fig. 5a–c and Extended Data Fig. 5d–g). We then used the regulatory network to test whether homologous genes were induced in *Arabidopsis*. We found that *Arabidopsis LRP1*, *MYB4* and *PAT1* were all induced during *Arabidopsis* grafting, suggesting a broadly conserved regulatory response between *Arabidopsis* and *Picea abies* (Fig. 5c and Extended Data Fig. 5d,g). As the *PAT1* gene family promotes root tip regeneration^26,28^, we focused on this family and tested an *Arabidopsis PAT1* overexpression line (*AtPAT1OE*) and *pat1*-related mutants in callus formation assays as callus is relevant for graft healing^12,27,39^. The *AtPAT1OE* line showed increased callus formation at the wounding sites of petioles, while *pat1*, *pat1scl5* and *pat1scl5scl21* showed strong impairment in callus formation. *scl5* showed mild defects in callus formation, whereas *scl21* did not show major differences compared to wild type (Col-0) (Fig. 5d–f). To investigate this gene family in *Picea abies*, we first constructed a phylogenetic tree with *PaPAT1-like* and *Arabidopsis* GRAS family genes. The phylogeny indicated that *PaPAT1-like* was most closely related to *AtPAT1*, *AtSCL5* and *AtSCL21*. An amino acid alignment also showed strong similarity between proteins of these genes (Extended Data Fig. 6a–c). To examine whether *PaPAT1-like* could perform a similar function as *Arabidopsis PAT1*, we cloned the *Picea abies PaPAT1-like* gene to generate an inducible overexpression line in *Arabidopsis* (*PaPAT1-likeOE*). We found that *PaPAT1-likeOE* increased callus area in cut petioles compared to wild type (Col-0) (Fig. 5g). ERF115 can interact with PAT1 family genes, and *AtERF115* and *PaERF115* were upregulated in both *Arabidopsis* and *Picea abies* transcriptomes (Extended Data Fig. 5h). We cloned and overexpressed *PaERF115-like* (*PaERF115-likeOE*) in the *Arabidopsis AtPAT1OE* background and found *PaERF115-like AtPAT1OE* massively increased callus formation similar to *AtERF115OE AtPAT1OE* (Extended Data Fig. 7c). Next, to investigate the role of *PAT1* in grafting, we tested *Arabidopsis* graft attachment rates and found *pat1scl5*, *scl5scl21*, *pat1scl21* and *pat1scl5scl21* all reduced attachment rates (Fig. 5h). In CFDA-mediated phloem reconnection assays, the *PAT1OE* line showed no changes, but the single, double and triple *AtPAT1*-related loss of function mutants all showed moderate to strong inhibition of phloem reconnection (Fig. 5i) including when combined with *erf115* mutants (Extended Data Fig. 7a,b). We also found that *PaPAT1-likeOE* could partially rescue the graft attachment defect of *pat1scl5* and *scl5scl21* double mutants (Fig. 5j). As our grafting assays used young hypocotyls, we also grafted with *Arabidopsis* inflorescence stems to look at the effects of age. However, we saw no evidence of *AtPAT1* induction at the inflorescence graft junction (Extended Data Fig. 8). Together, our results indicated that grafting-induced *PAT1* up-regulation was shared between *Arabidopsis* and *Picea abies* and that this gene has a conserved role in wound healing in juvenile tissues.

## Discussion

Propagating and combining species through grafting is commonly used worldwide, highlighting the need to establish efficient and practical grafting methods for diverse species^8,39,40^. Here we developed a micrografting method that allowed multiple conifer species to be grafted together, expanding the range of compatible graft combinations and providing insights into grafts formation between *Picea*, *Pinus* and *Larix* members that are estimated to have diverged from each other over 100 million years ago^41^. Previous conifer micrografting methods between mature leaf and young seedling rootstocks had low grafting success rates and seasonal limitations^7^. In this study, our rapid micrografting approach (50 grafts per hour) provided high success rates in both homografted and inter-species conifer graft combinations. Inter-genus combinations also initially showed high survival frequency, although with time, many combinations died or showed stunted growth consistent with delayed incompatibility^42^. However, several inter-genus combinations were successful after several years. *Picea abies* rootstocks were compatible (20–33%) with *Pinus sylvestris*, *Pinus contorta* and *Larix sibirica* scions. Reciprocal grafts with *Picea abies* scions had much lower long-term success rates suggesting *Picea abies* rootstocks allowed inter-genus graft compatibility, whereas scions did not. Previous inter-genus grafts between *Lotus japonicus* and *Medicago truncatula*, or *Solanum lycopersicum* and *Capsicum annuum*, also showed different efficiencies when scion and rootstock genotypes were reversed^35,43^. The basis for this ‘graft polarity’ is not well known, but *Arabidopsis alf4* mutants deficient in auxin response perturb grafting in the rootstock but not in the scion, consistent with *Arabidopsis* rootstocks being more sensitive to auxin than scions^17^ and suggesting at least one plausible explanation for differences in grafting efficiencies between scion and rootstock being related to auxin responsiveness.

Our transcriptome analysis of the *Picea abies* graft junction revealed dynamic activation of genes related to cell division, cambial identity, phloem and xylem, similar to what has been previously described at the *Arabidopsis* graft junction (Fig. 3 and Extended Data Fig. 3)^23^. However, there were notable differences. In *Arabidopsis*, phloem reconnection (3–4 DAG) is followed by xylem reconnection (6–7 DAG)^17^, whereas our *Picea* transcriptomes showed the activation of putative xylem and phloem markers at similar times, although our time course was limited as the *Arabidopsis thaliana* experiments had better temporal resolution given that graft healing took a longer time in *Picea* and *Pinus*. However, we found multiple similarities regarding the physiology and gene expression changes during grafting, pointing to a conserved process. Our study also identified auxin-responsive genes in *Picea abies* and found elevated auxin response at the graft junction that correlated with the activation of cell-wall-related genes (Fig. 3 and Extended Data Fig. 4). Both auxin and cell-wall-related processes promote graft formation in *Arabidopsis*, grape, *Nicotiana* and tomato^23,38,44,45^, implying that auxin response and cell wall modifications are important for graft formation in both eudicots and conifers.

Our gene regulatory network analysis identified several expression hubs including a *PaPAT1-like* homolog of an *Arabidopsis* GRAS transcription, *PAT1*. The PAT1 protein sequence was highly similar between *Picea* and *Arabidopsis*, in particular the C-terminus found in many GRAS/SCL family members (>78% of protein sequence identity). Our cross-species complementation analysis showed that PaPAT1 was functional and acted similar to AtPAT1 as we observe partial rescue of graft attachment phenotypes and callus formation. Previous studies have also used genes from tree species to modify *Arabidopsis* function. MADS (for MCM1, AG, DEF and SRF) -box genes *DAL2*, *DAL11*, *DAL12* and *DAL13* from *Picea abies* showed a common function with homologous genes in angiosperms suggesting conservation of genes structure and activity^46,47^. A poplar *PeSCL7* can improve salt tolerance of *scl7* mutant in *Arabidopsis*^48^, whereas several conifer genes including *HBK1*, *SAG1* and *NEEDLY* can functionally substitute for their homologous genes in *Arabidopsis*^49–51^. Such heterologous techniques are useful tools to understand the function of these genes outside of their endogenous system, particularly with the limitations of Clustered Regularly Interspaced Short Palindromic Repeats (CRISPR) mutations in trees; however, further work is needed with *Picea abies* mutated in *PAT1* to confirm its endogenous role in wound healing and graft formation.

Our work with *Picea abies* helped identify *PAT1* as a novel regulator of tissue attachment and graft formation in *Arabidopsis*. We also see evidence that a *PAT1* homolog is induced by wounding in rice (Extended Data Fig. 7d)^16^. Monocot grafting succeeds due to the use of embryonic tissues^16^, and here, the use of young tissues also allowed divergent conifer grafts to succeed. *AtPAT1* expression was not induced by *Arabidopsis* inflorescence grafting or cutting (Extended Data Fig. 8) suggesting PAT1 might be a key factor giving juvenile tissues a higher regeneration competency. Our findings imply that grafting young tissues helps overcome incompatibility in both gymnosperms and angiosperms and present a useful tool to extend the range of successful grafting in seed plants.

## Methods

### Plant materials and growth conditions

Conifer genotypes used were as follows: *Picea abies*—FP-96 Skogsgård (for grafting and generating graft junction libraries) and FP-518 TreO G7 Söregärde (for generating hormone treatment libraries); *Pinus sylvestris*—FP-601 Almnäs; *Pinus contorta*—FP-704 Lögdö; *Larix* hybrid (*Larix × Marschlinsii*)—FP-73 Långtora; and *Larix sibirica*—SV309 Lassinmaa. All conifer seeds were surface sterilized in 30% hydrogen peroxide solution for 20 min, followed by three rinses in autoclaved water; the sterilized seeds were imbibed overnight in darkness. The seeds were germinated on 1/4 Murashige and Skoog (MS) medium + 1% sucrose + 1% phytagel and stored vertically in a dark growth cabinet for 6 days; the seedlings were then moved to a low light condition (covered with a A4 paper) for an additional 1 day, and then seedlings were grown in a plant growth chamber (16/8 h light/dark, ∼110 μmol m^−2^ s^−1^, 22 °C, chamber). Ten- to twelve-day-old seedlings were used for micrografting. The grafted plants were grown in the same plant growth chamber in 2 months after grafting, followed by growing for 1 year in a greenhouse, then a subset of the plants was moved to grow in a field at the Skogforsk research station, Ekebo, Sweden.

All *A. thaliana* mutants used in this study were in the Columbia-0 background. *p35S::ERF115*(*AtERF115OE*), *p35S::PAT1*(*AtPAT1OE*), *pPAT1:NLS–GFP/GUS, pat1*, *erf115*, *scl5*, *scl21*, *erf115pat1*, *pat1scl5*, *scl5scl21*, *pat1scl21*, *erf115scl5* and *pat1scl5scl21* were previously published^26,28^. To generate *p35S::XVE»PaERF115-like-YFP* (*PaERF115-likeOE*) and *p35S::XVE»PaPAT1-like-YFP* (*PaPAT1-likeOE*), the open reading frame sequence without stop of *PaERF115-like* and *PaPAT1-like* were amplified from *Picea abies* complementary DNA (cDNA) and cloned into *pDONR221*. RNA extraction used the cetyltrimethylammonium bromide (CTAB)/lithium chloride (LiCl) method^52^ (‘RNA extraction’); cDNA was synthesized using a First Strand cDNA Synthesis Kit (K1612, Thermo Fisher Scientific). The primers used for cloning spruce genes are listed in Supplementary Table 6. All three segments of the entry vectors carrying the promoter of *p35S::XVE*^53^, followed by the entry vector carrying the open reading frame and the yellow fluorescent protein (YFP) with terminator, were combined into destination vector *pFR7M34GW*^54^. The constructs were transformed into GV3101 *Agrobacterium* competent cell for plant transformation. Seeds were surface sterilized for 10 min in 70% ethanol, followed by a rinse with 99.5% ethanol, air-dried and sown on petri dishes containing 1/2 MS medium + 0.8 % (*w*/*v*) agar. Seeds were stratified at 4 °C for 48 h, then moved to plant growth chamber (8/16 h, light/dark, 80–100 μmol m^−2^ s^−1^ light intensity) and grown vertically.

### Plant micrografting and measurement of attachment and vascular reconnection

*A. thaliana* micrografting and CFDA (VWR International) assays for measuring vascular reconnection were performed according to previously published protocols^17^. For testing graft attachment, we picked up the cotyledon and root of grafted plants with forceps. Unseparated grafts were counted as attached. For phloem assays, the CFDA was dropped on a cotyledon which was wounded by pressing with forceps. After 1 h, fluorescence was monitored in the rootstock as an indication of phloem connectivity. Attachment and phloem reconnection were both checked 3 DAG.

For conifer micrografting, 10- to 12-day-old plants were excised in the hypocotyl region 0.5 cm below the needles. Scions and rootstocks from different plants were attached tightly together using a 0.8 mm inner diameter silicon collar. To check for the phloem reconnection, we removed all leaves and dipped the cut site into 1 mM CFDA solution. Two hours after applying, we made a hand section of the rootstocks to observe the CFDA fluorescence. For xylem reconnection assays, we removed the tissues 1 cm below the graft junction, dipping the cut part into 5 μl CFDA for 2 h, then observed the fluorescence in the needles. To look for xylem differentiation at the junction, we made longitudinal sections at the graft junction, cleared the sections with acidified methanol (methanol:37% HCl:H_2_O = 10:2:38)^55,56^ and incubated at 55–57 °C for 15–20 min. We then replaced the clearing buffer with 7% NaOH in 60% ethanol and incubated them for 15 min at room temperature, followed by rehydration with 40%, 20% and 10% ethanol, incubating for 15 min in each ethanol solution. The rehydrated sections were stained with 0.01% basic fuchsin solution (dissolved in water) for 5 min. The staining was stopped with 70% ethanol for 15 min, and sections were rehydrated with 10% ethanol for 15 min, before adding 50% glycerol and incubating for 30 min. Sections were mounted in 50% glycerol for imaging.

### *Arabidopsis* inflorescence stem grafting and incisions

The inflorescence stem grafting was performed following a previously published method^57^. Grafting was performed using a wedge graft method when the primary inflorescence meristem had grown to 10 cm, leaving the stock around 3 cm above the rosette. The graft junction was sealed by wrapping with parafilm. The grafted plants were growing in a clear plastic box to maintain a humid environment. The stem incision experiment was performed according to a published method^58^. Using a microsurgical knife (Surgical Specialties), a mature flowering stem was incised approximately 3 cm from its base, reaching halfway through its diameter.

### Imaging

Grafted plants were imaged using a Nikon D5300 camera. The CFDA fluorescence was observed with a Leica M205 FA stereofluorescent microscope fitted with a YFP filter. Basic fuchsin-stained samples and β-glucuronidase (GUS)-stained samples were imaged with a Zeiss Axioscope A1 microscope or M205 FA stereofluorescent microscope. The higher-resolution images of hand sections were imaged with an LSM-780 confocal microscope. For CFDA imaging, 488 nm excitation and 500–560 nm emission settings were used. Wavelengths of 561 nm excitation and 571–690 nm emission were used for imaging basic fuchsin-stained samples. All images were analysed using Zen blue or Fiji (version 2.9.0/1.53t)^59^.

### Hormone treatment

Two-week-old *Picea abies* seedlings were selected for hormonal treatment. *Picea abies* seedlings were treated with either BAP, IAA or a combination of BAP and IAA hormones. The hormone concentrations were BAP 135 mM, IAA 50 mM, BAP 135 mM + IAA 50 mM. Approximately 20 μl of hormone dissolved in 70% ethanol was quickly mixed with a small amount of lanolin. The hormone lanolin paste was then applied to the middle hypocotyl region of the seedlings, approximately 1.5 cm below the needles. The treated area (8–10 mm in length) was wrapped in aluminium foil.

### Construction and analysis of RNA-seq libraries

#### Graft junction libraries

Samples were collected from both ungrafted (intact) and grafted *Picea abies* above and below the graft junction at 0, 1, 3, 7, 14 and 28 DAG. Approximately 1 mm of tissues from scions or rootstocks or 2 mm from intact plants was collected. Samples were collected in three biologically independent replicates for all treatments. Five plants were pooled for each replicate.

#### Hormone treatment libraries

Samples were collected for analysis after 5, 10 and 15 days. Approximately 5 mm of the treated hypocotyl was collected from each seedling, and the sample was snap frozen with liquid nitrogen. Samples were collected in three biologically independent replicates for all treatments. Eleven plants were pooled for each replicate.

#### RNA extraction

Total RNA was extracted using a modified CTAB/LiCl method^52^. Briefly, frozen tissue was ground into a fine powder. Extraction buffer was prepared (100 mM Tris–HCl (pH 8), 2% (*w*/*v*) CTAB, 30 mM EDTA, 2 M NaCI, 0.05% (*w*/*v*) spermidine, 2% (*w*/*v*) PVPP, 2% (*v*/*v*) 2-mercaptoethanol, proteinase K (10 mg ml^−1^) to a final concentration of 1.5 mg ml^−1^) and warmed for 10 min at 42 °C, then added to the ground frozen tissue and incubated at 42 °C for 90 min. Chloroform-isoamyl alcohol (24:1 (*v*/*v*)) was added to extract RNA. After vortexing and centrifuging at 15,000 *g* for 15 min at 4 °C, the top aqueous phase was transferred to a new tube, and 1/4 volume of 10 M LiC1 was added, allowing overnight precipitation at 4 °C. Samples were centrifuged at 15,000 *g* for 30 min and the supernatant discarded. Finally, the pellet was washed with 2 M LiCl twice and dissolved in RNase-free water. RNA concentration was measured using Qubit 2.0. The RNA integrity number was analysed by using an Agilent 2100 Bioanalyzer with RNA 6000 Nano kit. The RNA integrity numbers of all samples were above 8.0.

#### Libraries construction

About 1 µg total RNA per sample was used for RNA-seq library preparation. For grafting junction libraries, we followed the New England Biolabs (NEB) library building method. mRNA isolation was performed using NEBNext Poly(A) mRNA Magnetic Isolation Module (NEB number E7490S), followed directly by using NEBNext Ultra Directional RNA Library Prep Kit for Illumina (NEB number E7760S) and NEBNext Multiplex Oligos for Illumina (NEB number E7600S) for library construction. The quality of DNA libraries was checked with an Agilent 2100 Bioanalyzer DNA High Sensitivity Kit. The hormone treatment libraries were performed by Novogene (UK).

#### Bioinformatic analysis

Sequencing was performed on Illumina NovaSeq 6000 system with PE150. RNA-seq analysis was performed as previously described with minor modifications^60^. Briefly, the quality of raw data was accessed using FastQC (http://www.bioinformatics.babraham.ac.uk/projects/fastqc/) (version 0.11.8). The residual rRNA contamination was removed using SortMeRNA (version 4.3.3)^61^. Data were then filtered using fastp (version 0.20.0)^62^. After both filtering steps, FastQC was run again to ensure that no technical artefacts were introduced during the pre-processing steps. Filtered reads were aligned to version 1.0 of the *Picea abies* genome (retrieved from the PlantGenIE, https://plantgenie.org/FTP?dir=Data%2FPlantGenIE%2FPicea_abies%2Fv1.0) using STAR (version 2.7.9a)^63^. The parameters of RNA-seq data pre-processing followed the previously described guideline^60^. Read counts were quantified by HTSeq^64^ using *Picea abies* version 1.0 GFF file (retrieved from the PlantGenIE), with setting -s reverse. DEGs were identified using the DESeq2 package (version 3.13) in R (version 4.0.4)^65^. In each time point during grafting, intact samples were used as a reference. However, for intact samples, day 0 was the reference, while for hormone treated samples, mock treatment was the reference. Genes with an absolute log_2_(fold change) value above 1 and a *q* value below 0.05 were considered differentially expressed. The normalized reads obtained from DEseq2 were used for gene expression. The analysis of common patterns of gene expression during grafting was performed using the Mfuzz package (version 3.15) in R^66^. First, DEGs of grafted tissues (scion and rootstock) from all time points were combined into a list to consider for analysis. Then based on the DEGs list, day 0 intact samples were also included to identify the common gene expression patterns during grafting. GO enrichment analysis of clusters was performed by hypergeometric distribution in R, with an adjusted *P* < 0.05 and fold change >2 as the cut-off to determine significantly enriched GO terms. We downloaded the spruce GO annotation file from the PlantGenIE. For the overlap analysis, overlap was presented as a ratio of 1.0 for DEGs up- or down-regulated in the grafting dataset relative to intact samples compared with up- and down-regulated genes in the hormone datasets relative to mock. The gene co-expression network was conducted using the WGCNA package (version 1.71)^67^ in R. Only 524 putative transcriptional factors that differentially expressed during grafting were analysed. To increase the sample size, the individual replicates were introduced as one sample. Then a module that showed positive regulation during the grafting was considered to discover the hubs of regulatory interactions. Then the interactions were visualized in Cytoscape 3 (version 3.9.1)^68^. The orthologs between *Picea abies* and *Arabidopsis* were obtained from pabies_artha.tsv (retrieved from the PlantGenIE, https://plantgenie.org/FTP?dir=Data%2FCross-Species%2FOrthologs). The list of DEGs from all comparisons and the orthologs, and transcription factor of the regulatory network are provided in Supplementary Table 7, as well as the genes used for heat map plots.

#### Quantitative reverse transcription-PCR (qRT-PCR) assays

Samples were collected from *Arabidopsis* inflorescence stems, from both grafted and non-grafted plants. Total RNA was extracted using a Roti-Prep RNA MINI Kit. About 500 ng RNA was used for cDNA synthesis using the Maxima First Strand cDNA Synthesis Kit (Thermo Fisher Scientific), which includes oligo(dT)18 primers. Quantitative reverse transcription-PCR (qRT-PCR) reaction was performed with 2× Maxima SYBR Green qPCR/ROX Master Mix (Thermo Fisher Scientific) and run with a Bio-Rad CFX96 qPCR machine. The results were analysed using the 2-^ΔΔCT^ method. The primers used for quantitative reverse transcription PCR are in Supplementary Table 6.

#### Callus induction

Cotyledons with petioles excised from 10-day-old seedlings growing in long-day conditions were used for callus induction^27^. The cotyledon explants were placed on petiole callus induction medium (MS) medium plates supplemented with 1% sucrose and 0.6% Gelrite) and induced for 7 days under long-day conditions. For callus formation without wounding, the F1 generation of *AtERF115OE AtPAT1OE* seedlings were grown on 1/2 MS medium for 21 days, and the T2 generation of *PaERF115-likeOE AtPAT1OE* seedlings were grown on 1/2 MS medium with 10 µM estradiol (Sigma-Aldrich) for 7 days, then transferred to 1/2 MS medium for 14 days.

#### Estradiol treatment

For callus induction and grafting assay using *Arabidopsis*, plants growing medium and callus induction medium were both prepared containing 10 μM estradiol (Sigma-Aldrich). The grafted plants were placed on the filter paper containing water with 10 μM estradiol.

#### Phylogenetic analysis and amino acid alignment

The protein sequence of *Arabidopsis* GRAS family and spruce *PaPAT1-like* were used for phylogenetic analysis with MEGA11 software (version 11.0.13)^69^. The protein sequences were aligned by ClustalW (offered in MEGA11); Construct/Test neighbour-joining tree (offered in MEGA11) was used to estimate phylogenetic trees. Bootstrap replicates number was set to 1,000. Amino acid alignment was generated with Snapgene software (version 5.1.4.1; www.snapgene.com). The protein similarity and heat map was generated using TBtools software (version 1.120)^70^.

#### GUS staining

Plant tissues were submerged in cold acetone for 10 min. After washing with staining solution without x-gluc, tissues were transferred into staining solution (100 mM sodium phosphate buffer pH 7.0, 10 mM EDTA, 1 mM K_3_[Fe(CN)_6_], 1 mM K4[Fe(CN)_6_], 2 mM 5-bromo-4-chloro-3-indolyl-ß-glucuronide) and vacuum infiltrated for 10 min at room temperature. Tissues were incubated in the staining solution for 2 h at 37 °C. Stained tissues were submerged in 70% ethanol until the GUS was cleared.

#### Measurement of plant size

For plant height measurements, the shoot length of the above-ground part was measured as the plant height. For the shoot diameter measurements, the shoot diameter of grafted plants was measured at 3 cm above the graft, and the shoot diameter of intact plants was measured at a site similar to grafted plants.

#### Statistical analysis

Statistical analyses methods were used as indicated in the figure legends. Student’s *t*-test was performed with Excel (version 16.72), Wilcoxon signed-rank test and one-way analysis of variance (ANOVA) followed by Tukey honestly significant difference test were performed with R (version 4.0.2).

### Reporting summary

Further information on research design is available in the Nature Portfolio Reporting Summary linked to this article.

### Supplementary information

Supplementary Information Legends of Supplementary Tables 1–7.

Reporting Summary

Supplementary Table 1 Survival rate of plants 2 months after grafting.

Supplementary Table 2 Plant height 2.5 years after grafting.

Supplementary Table 3 Differentially expressed genes in grafted *Picea abies* junction and intact plants.

Supplementary Table 4 Go enrichment analysis of co-expressed clusters.

Supplementary Table 5 Differentially expressed genes of auxin and cytokinin treatments.

Supplementary Table 6 Primers used for cloning spruce genes and qRT-PCR.

Supplementary Table 7 Gene IDs of analysed spruce genes.

### Source data

Source Data Fig. 1 Statistical source data.

Source Data Fig. 2 Statistical source data.

Source Data Fig. 3 Statistical source data.

Source Data Fig. 4 Statistical source data.

Source Data Fig. 5 Statistical source data.

Source Data Table 1 Statistical source data.

Source Data Extended Data Fig. 2 Statistical source data.

Source Data Extended Data Fig. 3 Statistical source data.

Source Data Extended Data Fig. 4 Statistical source data.

Source Data Extended Data Fig. 5 Statistical source data.

Source Data Extended Data Fig. 7 Statistical source data.

Source Data Extended Data Fig. 8 Statistical source data.

## Data Availability

All data are available in the manuscript, supplementary information, extended data and source files. Transcriptome data reported in this paper are deposited in the NCBI Gene Expression Omnibus (GEO) database under the accession number GSE231633. Spruce genome information and orthologs were retrieved from the PlantGenIE site (https://plantgenie.org/FTP?dir=Data%2FplantGenIE%2Fpicea_abies%2Fv1.0). *OsPAT1-like* (Os07g0583600) expression values were obtained from a previously published dataset^16^. Source data are provided with this paper.
