# Supplementary material for: A conserved graft formation process in Norway spruce and Arabidopsis identifies the PAT gene family as central regulators of wound healing
Source: Nat Plants. 2024 Jan 2;10(1):53–65. doi: 10.1038/s41477-023-01568-w (PMC10808061; doi:10.1038/s41477-023-01568-w)
Supplement: Supplementary file 1 — Legends of Supplementary Tables 1–7. [file 41477_2023_1568_MOESM1_ESM.pdf]

nature plants

Article https://doi.org/10.1038/s41477-023-01568-w

A conserved graft formation process in Norway spruce and *Arabidopsis* identifies the PAT gene family as central regulators of wound healing

In the format provided by the authors and unedited

Legends of supplementary tables

Supplementary Table 1. Survival rate of plants two months after grafting.

Supplementary Table 2. Plant height 2.5 years after grafting.

Supplementary Table 3. Differentially expressed genes in grafted *Picea abies* junction and intact plants.

Supplementary Table 4. Go enrichment analysis of co-expressed clusters.

Supplementary Table 5. Differentially expressed genes of auxin and cytokinin treatment.

Supplementary Table 6. Primers used for cloning spruce genes and qRT-PCR.

Supplementary Table 7. Gene IDs of analysed spruce genes.
