# Supplementary material for: A conserved graft formation process in Norway spruce and Arabidopsis identifies the PAT gene family as central regulators of wound healing
Source: Nat Plants. 2024 Jan 2;10(1):53–65. doi: 10.1038/s41477-023-01568-w (PMC10808061; doi:10.1038/s41477-023-01568-w)
Supplement: Supplementary file 2 — Reporting Summary [file 41477_2023_1568_MOESM2_ESM.pdf]

nature portfolio

Corresponding author(s): Charles W. Melnyk

Last updated by author(s): October 4, 2023

# Reporting Summary

Nature Portfolio wishes to improve the reproducibility of the work that we publish. This form provides structure for consistency and transparency in reporting. For further information on Nature Portfolio policies, see our Editorial Policies and the Editorial Policy Checklist.

## Statistics

For all statistical analyses, confirm that the following items are present in the figure legend, table legend, main text, or Methods section.

| n/a | Confirmed | |
|---|---|---|
| ☐ | ☒ | The exact sample size (*n*) for each experimental group/condition, given as a discrete number and unit of measurement |
| ☐ | ☒ | A statement on whether measurements were taken from distinct samples or whether the same sample was measured repeatedly |
| ☐ | ☒ | The statistical test(s) used AND whether they are one- or two-sided<br>*Only common tests should be described solely by name; describe more complex techniques in the Methods section.* |
| ☒ | ☐ | A description of all covariates tested |
| ☐ | ☒ | A description of any assumptions or corrections, such as tests of normality and adjustment for multiple comparisons |
| ☐ | ☒ | A full description of the statistical parameters including central tendency (e.g. means) or other basic estimates (e.g. regression coefficient) AND variation (e.g. standard deviation) or associated estimates of uncertainty (e.g. confidence intervals) |
| ☐ | ☒ | For null hypothesis testing, the test statistic (e.g. *F*, *t*, *r*) with confidence intervals, effect sizes, degrees of freedom and *P* value noted<br>*Give P values as exact values whenever suitable.* |
| ☒ | ☐ | For Bayesian analysis, information on the choice of priors and Markov chain Monte Carlo settings |
| ☒ | ☐ | For hierarchical and complex designs, identification of the appropriate level for tests and full reporting of outcomes |
| ☒ | ☐ | Estimates of effect sizes (e.g. Cohen's *d*, Pearson's *r*), indicating how they were calculated |

*Our web collection on statistics for biologists contains articles on many of the points above.*

## Software and code

Policy information about availability of computer code

| | |
|---|---|
| Data collection | We used commercially available softwares Image J (Version 2.9.0/1.53t ) and Zen Blue to record and analyse data from microscopes. RNA-Seq data was acquired by a Illumina NextSeq sequencer. qRT-PCR data was collected from a Bio-Rad CFX96 qPCR machine. |
| Data analysis | R(version 4.0.4) was used for transcriptome analysis and generating plots. The quality of raw data was accessed using FASTQC (Version 0.11.8) (http://www.bioinformatics.babraham.ac.uk/projects/fastqc/). The residual rRNA contamination was removed using SortMeRNA (version 4.3.3)(Kopylova et al. 2012). Data were then filtered using fastp (version 0.20.0) (Chen et al., 2018). After both filtering steps, FASTQC was run again to ensure that no technical artifacts were introduced during the pre-processing steps. Filtered reads were aligned to version 1.0 of the Norway spruce genome (retrieved from the PlantGenIE) using STAR (version 2.7.9a) (Dobin et al., 2013). The parameters of RNA-seq data pre-processing followed the previously described guideline(Delhomme et al., 2014). Read counts were quantified by HTSeq (version 2.0.1) (Anders et al., 2015) using Norway spruce version 1.0 GFF file (retrieved from the PlantGenIE), with setting -s reverse. Differentially expressed genes (DEGs) were identified using the DESeq2 package (version 3.13) in R. The analysis of common patterns of gene expression during the grafting was performed using the Mfuzz package (version 3.15) in R. The gene co-expression network was conducted using the WGCNA package (version 1.71). Cytoscape 3 (version 3.9.1) were used to visualize the regulatory interactions.<br>Excel (version 16.72) was for Student's t test analysis and making plots.<br>MEGA (version 11.0.13) for the phylogenetic tree.<br>TBtools (version 1.120) for generating heatmaps and calculating protein sequence similarity.<br>Snapgene (version 5.1.4.1) for alignment of protein sequence. |

For manuscripts utilizing custom algorithms or software that are central to the research but not yet described in published literature, software must be made available to editors and reviewers. We strongly encourage code deposition in a community repository (e.g. GitHub). See the Nature Portfolio guidelines for submitting code & software for further information.

## Data

Policy information about availability of data

All manuscripts must include a data availability statement. This statement should provide the following information, where applicable:

- Accession codes, unique identifiers, or web links for publicly available datasets
- A description of any restrictions on data availability
- For clinical datasets or third party data, please ensure that the statement adheres to our policy

All RNA-seq data has been uploaded into NCBI. The Gene Expression Omnibus (GEO) accession number for the transcriptomic data reported in this paper is GEO: GSE231633. This study did not generate original code. The Picea abies genome orthologs (pabies_artha.tsv) was retrieved from PlantGenIE (https://plantgenie.org/FTP?dir=Data%2FPlantGenIE%2FPicea_abies%2Fv1.0).

## Research involving human participants, their data, or biological material

Policy information about studies with human participants or human data. See also policy information about sex, gender (identity/presentation), and sexual orientation and race, ethnicity and racism.

| | |
|---|---|
| Reporting on sex and gender | not applicable |
| Reporting on race, ethnicity, or other socially relevant groupings | not applicable |
| Population characteristics | not applicable |
| Recruitment | not applicable |
| Ethics oversight | not applicable |

Note that full information on the approval of the study protocol must also be provided in the manuscript.

# Field-specific reporting

Please select the one below that is the best fit for your research. If you are not sure, read the appropriate sections before making your selection.

☒ Life sciences ☐ Behavioural & social sciences ☐ Ecological, evolutionary & environmental sciences

For a reference copy of the document with all sections, see nature.com/documents/nr-reporting-summary-flat.pdf

# Life sciences study design

All studies must disclose on these points even when the disclosure is negative.

| | |
|---|---|
| Sample size | Sample size was determined according to similar studied in the field. Reference: Thomas, H., Van den Broeck, L., Spurney, R., Sozzani, R. & Frank, M. Gene regulatory networks for compatible versus incompatible grafts identify a role for SlWOX4 during junction formation. Plant Cell 34, 535-556 (2022). Sample size was also selected based on our prior knowledge and experience on the experimental variability of experiments and the desire to get statistically significant data to support meaningful conclusions. This resulted sample sizes were from n=3 to n=73 to ensure the variation was captured. Sample sizes were described in figure legends or in the main text. |
| Data exclusions | No data were excluded from this study. |
| Replication | All experiments were repeated at least two independent times. Plants growing in long term (>2 years) field experiments were performed with multiple plants (6-34 plants per combination) grown as one field plot. |
| Randomization | Seedlings or samples were randomly distributed during growth and treatments |
| Blinding | Data were collected according to the plant genotypes and treatments. Blinding experiments were performed with RNA extractions and library preparations. |

# Reporting for specific materials, systems and methods

We require information from authors about some types of materials, experimental systems and methods used in many studies. Here, indicate whether each material, system or method listed is relevant to your study. If you are not sure if a list item applies to your research, read the appropriate section before selecting a response.

## Materials & experimental systems

| n/a | Involved in the study | |
|---|---|---|
| ☒ | ☐ | Antibodies |
| ☒ | ☐ | Eukaryotic cell lines |
| ☒ | ☐ | Palaeontology and archaeology |
| ☒ | ☐ | Animals and other organisms |
| ☒ | ☐ | Clinical data |
| ☒ | ☐ | Dual use research of concern |
| ☐ | ☒ | Plants |

## Methods

| n/a | Involved in the study | |
|---|---|---|
| ☒ | ☐ | ChIP-seq |
| ☒ | ☐ | Flow cytometry |
| ☒ | ☐ | MRI-based neuroimaging |
